# Thermal Stability Analysis of Lithium-Ion Battery Electrolytes Based on Lithium Bis(trifluoromethanesulfonyl)imide-Lithium Difluoro(oxalato)Borate Dual-Salt

**DOI:** 10.3390/polym13050707

**Published:** 2021-02-26

**Authors:** Ya-Ping Yang, An-Chi Huang, Yan Tang, Ye-Cheng Liu, Zhi-Hao Wu, Hai-Lin Zhou, Zhi-Ping Li, Chi-Min Shu, Jun-Cheng Jiang, Zhi-Xiang Xing

**Affiliations:** 1 School of Material Science and Engineering, Changzhou University, Changzhou 213164, China; B1900012@smail.cczu.edu.cn (Y.-P.Y.); B20080526@smail.cczu.edu.cn (Y.-C.L.); 2 School of Environmental and Safety Engineering, Changzhou University, Changzhou 213164, China; 19083700144@smail.cczu.edu.cn (Z.-H.W.); 19083700424@smail.cczu.edu.cn (H.-L.Z.); 19083700288@smail.cczu.edu.cn (Z.-P.L.); 3 Department of Safety, Health and Environmental Engineering, National Yunlin University of Science and Technology, Yunlin 64002, Taiwan; shucm@yuntech.edu.tw

**Keywords:** LiTFSI-LiODFB dual-salt carbonate electrolyte, thermal analysis, accelerated rate calorimetry, differential scanning calorimetry, autocatalytic models, apparent activation energy

## Abstract

Lithium-ion batteries with conventional LiPF_6_ carbonate electrolytes are prone to failure at high temperature. In this work, the thermal stability of a dual-salt electrolyte of lithium bis(trifluoromethanesulfonyl)imide (LiTFSI) and lithium difluoro(oxalato)borate (LiODFB) in carbonate solvents was analyzed by accelerated rate calorimetry (ARC) and differential scanning calorimetry (DSC). LiTFSI-LiODFB dual-salt carbonate electrolyte decomposed when the temperature exceeded 138.5 °C in the DSC test and decomposed at 271.0 °C in the ARC test. The former is the onset decomposition temperature of the solvents in the electrolyte, and the latter is the LiTFSI-LiODFB dual salts. Flynn-Wall-Ozawa, Starink, and autocatalytic models were applied to determine pyrolysis kinetic parameters. The average apparent activation energy of the dual-salt electrolyte was 53.25 kJ/mol. According to the various model fitting, the thermal decomposition process of the dual-salt electrolyte followed the autocatalytic model. The results showed that the LiTFSI-LiODFB dual-salt electrolyte is significantly better than the LiPF_6_ electrolyte in terms of thermal stability.

## 1. Introduction

The development of high-energy-density, long-cycle-life, and high-safety secondary lithium-based batteries is essential to meet the emerging needs of the electronics and automotive industry, and various energy storage systems [1,2]. Developing high-voltage and high-capacity cathode materials is an indispensable requirement for promoting high- energy-density lithium-ion batteries (LIB). When the cathode materials are constant, increasing the charge cut-off voltage of the battery system can effectively increase its actual capacity [3]. Nevertheless, when the voltage exceeds 4.5 V, the traditional commercially available hexafluorophosphate (LiPF_6_) carbonate electrolyte is prone to oxidation and decomposition. Then the side reaction between the cathode and the electrolyte is intensified, and the transition metal ions are eluted, resulting in a significant decrease in the specific capacity of the battery [4,5].

Additionally, commercial electrolytes have high volatility and flammability, with an operating temperature range of –20 to 55 °C. Above 55 °C, LiPF_6_ decomposes and produces LiF and PF_5_. PF_5_ has firm acidity, causing the ring-opening reaction of cyclic carbonate in the solvent, and generating some linear carbonates, which corrodes the cathode structure material. LiPF_6_ is sensitive to moisture and will react with trace moisture impurities in the electrolyte to generate a small amount of hydrofluoric acid (HF), which will be more severe at high temperatures. These safety issues severely restrict the development of lithium-ion batteries [6,7]. Therefore, to meet the requirements of high energy density and high safety in lithium-ion batteries, it is desirable to improve the stability of the electrolyte under high pressure and high temperature.

The performance of the electrolyte mainly depends on the lithium salt in the electrolyte. The anion of the lithium salt plays a crucial role in electrolyte stability and the formation of the solid electrolyte mesophase (SEI) layer. Thus, developing a more stable lithium salt is undoubtedly a critical approach to improving lithium-ion battery electrolyte safety [8]. According to reports, LiTFSI has the advantages of sound thermal and chemical stability, high thermal decomposition temperature (360 °C), insensitivity to moisture, high ionic conductivity, and wide electrochemical window (glassy carbon as the working electrode, 5.0 V vs. Li^+^/Li) [9,10,11]. Moreover, LiTFSI has many applications in novel batteries, which can form a stable SEI film on the graphite anode, effectively improving the dendrite problem on the lithium anode; therefore, LiTFSI is expected to substitute LiPF_6_ [12,13]. However, LiTFSI will cause severe corrosion to the cathode current collector aluminum foil at a voltage above 3.7 V when used alone, limiting its use [14,15]. The lithium salt-based electrolyte of LiODFB has a wider electrochemical window for aluminum foil [16]. Due to the combination of Al^3+^ and B–O groups, a sound thermal stability, dense protective film can be formed on the surface of Al [17,18]. A stable passivation layer formed on the cathode has been reported after adding LiODFB to the lithium salt LiTFSI-based electrolyte, which can effectively inhibit the corrosion of the aluminum foil LiTFSI [19,20].

Recently, Qinglei Wang and his team have systematically explored the application of LiTFSI-LiODFB dual-salt in lithium-ion electrolytes. The study found that compared with high-voltage (5 V vs. Li^+^/Li) Li/LiNi_0.5_Mn_1.5_O_4_ cells using LiPF_6_ electrolytes, the cells with LiTFSI_0.5_-LiODFB_0.5_ dual-salt electrolyte had excellent cycling stability and rate performance [21]. When the cathode was LiFePO_4_ or LiCoO_2_, the cells with the best ratio of LiTFSI_0.6_-LiODFB_0.4_ (25 °C) and LiTFSI_0.4_-LiODFB_0.6_ (60 °C) dual-salt electrolytes in solvents ethylene carbonate (EC) and ethyl methyl carbonate (EMC), and both had excellent cycling stability and rate performance compared with using LiPF_6_ electrolyte [19,20]. Zhen Geng et al. found that Li/LiCoO_2_ cells have a high capacity (2.4 mAh/cm^2^) and excellent cycling performance at elevated temperature up to 80 °C, using 0.8 M LiTFSI-0.2 M LiODFB-0.01 M LiPF_6_ electrolytes in EC and propylene carbonate (PC) solvents [22]. Hongfa Xiang et al. demonstrated LiTFSI_0.6_-LiBOB_0.4_ dual-salt electrolytes in EC-EMC (4:6 by mass%) have an excellent charge ability and cycling stability of LiLiNi_0.8_Co_0.15_Al_0.05_O_2_ (NCA) cells. The NCA cells can deliver a discharge capacity of 131 mAh/g and capacity retention of 80% after 100 cycles at the charging current density of 1.50 mA/cm^2^ with the dual-salt electrolyte [23]. Studies have shown that whether used in lithium-ion batteries or lithium metal batteries, the LiTFSI-LiODFB dual-salt electrolyte is superior to LiPF_6_-based electrolytes in cycling stability and rate capability under specific proportions and specific conditions. It is crucial to improve the energy and safety of the battery. In addition, the conductivity of the LiTFSI-LiODFB dual-salt electrolyte decreases with the increase in the amount of LiODFB added, and the LiODFB-based electrolyte shows the maximum conductivity (8.58 mS/cm) in the EC and dimethyl carbonate (DMC) binary solvent mixture [24].

Therefore, considering the conductivity, electro-chemical performance, and high- temperature performance of the dual-salt electrolyte, compared with other ratios of LiTFSI-LiODFB dual-salt carbonate electrolyte, the ratio of LiTFSI_0.6_-LiODFB_0.4_ double-salt electrolyte has a broader application prospect in EC and DMC solvents. However, studies have paid insufficient attention to the thermal behavior of electrolytes mixed with lithium salts at high temperatures [25,26], and the specific role of mixed lithium salts in improving the thermal stability of the electrolyte has yet to be analyzed [27,28,29]. Therefore, in this work, differential scanning calorimetry (DSC) and accelerated rate calorimetry (ARC) were utilized to compared thermal behaviors between the LiTFSI-LiODFB dual-salt electrolyte and the LiPF_6_ electrolyte in the solvent mixed by ethylene carbonate (EC) and dimethyl carbonate (DMC) [30]. Various thermokinetic models were adopted to calculate the kinetic parameters and simulate the thermal decomposition process of electrolytes based on LiTFSI-LiODFB dual-salt [31]. The findings of the current study could provide reference information on the thermal stability of dual-salt electrolytes.

## 2. Materials and Methods

### 2.1. Materials

Battery-grade EC and DMC solvents were purchased from Sigma-Aldrich (purity > 99%). Lithium salt LiPF_6_ was purchased from Aldrich (purity ≥ 99.99% trace metals basis). Battery-grade LiTFSI (purity > 98%) and LiODFB (purity > 99%) were purchased from Adamas. All untreated chemicals were stored in a glove box filled with purified argon during the preparation of electrolytes. The dual-salt electrolyte was composed of 0.6 M LiTFSI and 0.4 M LiODFB (or LiTFSI_0.6_-LiODFB_0.4_) in EC+DMC (2:3, *v*/*v*). For comparison, the control electrolyte composed of 1 M LiPF_6_ in the same EC+DMC (2:3, *v*/*v*) mixture was investigated as well. The physico-chemical properties of the above electrolyte lithium salts and solvents are listed in Table 1.

### 2.2. Differential Scanning Calorimetry (DSC) Measurement

The DSC can measure the temperature and heat flow of the electrolyte sample under different atmospheres and heating rates related to the material conversion [32,33]. The heat-flow DSC 3 (produced by Mettler Toledo Co., Greifensee, Switzerland) was used to acquire the thermodynamic behavior of the self-made electrolyte. The matching standard aluminum crucible (40 µL) was selected to seal the electrolyte sample in the glove box to prevent the sample from contact with air and moisture. The DSC sample crucible was weighed before and after loading the sample, and the net sample mass was controlled within 3.5–5.0 mg. For the dynamic experiments, N_2_ (90 mL/min) atmosphere was employed, and the heating range was from 40 to 350 °C. Ten sets of samples for two electrolytes were scanned at different heating rates (*β*, *β =* 1, 2, 4, 7, and 10 °C/min) to obtain the vital thermodynamic parameters such as the onset temperature (*T*_o_), peak temperature (*T*_p_), end temperature (*T*_e_), and heat of reaction (Δ*H*) in entire pyrosis process [34].

### 2.3. Accelerated Rate Calorimetry (ARC) Measurement

As is known, the DSC 3 is an external heat-flow instrument, so it cannot directly reflect the actual reaction process of the material in an adiabatic environment. It also lacks the ability to detect the crucible pressure, so the pressure change of the material during the thermal runaway process cannot be obtained. Due to these limitations, it is necessary to further employ an accelerated rate calorimeter (ARC 244 from Netzsch, Selb, Germany) to measure the temperature and pressure changes of the electrolyte under pseudo adiabatic conditions [35]. In an argon atmosphere glove box, the titanium bomb was filled with LiPF_6_ and LiTFSI-LiODFB electrolyte samples for ARC experiments. In the adiabatic experiment, the ARC Hastelloy ball was heated to 120 °C. The heat-wait-search mode was initiated, then stopped when the temperature reached 350 °C. A heat-wait-search procedure was applied for every 5.0 °C increment with a waiting time of 15 min before detecting an exothermal reaction. When the heat generation rate of the sample exceeded 0.02 °C/min, the exotherm will be created. If no exotherm was found, the temperature increased with a heating rate of 10 °C/min [36]. In addition to the temperature and the heating rate, the pressure can also be recorded, and the self-temperature and self-pressure will also be calculated.

### 2.4. Kinetic Analysis

In a multivariate kinetic reaction, the activation energy (*E*_a_) is an apparent value related to temperature. The lower the *E*_a_ value, the more easily the reaction takes place. In this work, based on the thermodynamic parameters recorded from DSC experiments, model-free methods including Starink (the differential method) and Flynn-Wall-Ozawa (FWO, the integral method) were utilized to calculate the *E*_a_ of thermal decomposition of electrolytes [37,38].

#### 2.4.1. Starink Method

The Starink method is highly accurate and widespread, as offered in the following equation:

(1) $$\ln\left(\frac{\beta }{T^{1.8}}\right)=C_{S}-1.0037\frac{E_{a}}{RT}$$

where *C_s_* is a constant.

#### 2.4.2. FWO Method

In the FWO method, *E*_a_ can be calculated directly without the reaction mechanism function, thereby virtually eliminating the errors caused by mechanism functions. The FWO kinetic equation is shown as follows [39]:

(2) $$\mathrm{lg}\beta =\mathrm{lg}\left(\frac{AE_{a}}{RG\left(\alpha \right)}\right)-2.315-0.4567\frac{E_{a}}{R}\frac{1}{T}$$

In the same conversion rate, temperature *T* was taken of each thermal analysis curve with different *β*, linearly fitting lg*β* and 1/*T*. Then, the *E*_a_ was calculated from the slope of the straight line.

## 3. Results and Discussion

### 3.1. Thermal Analysis Technology

#### 3.1.1. Thermal Decomposition Analysis by DSC

**Figure 1:**
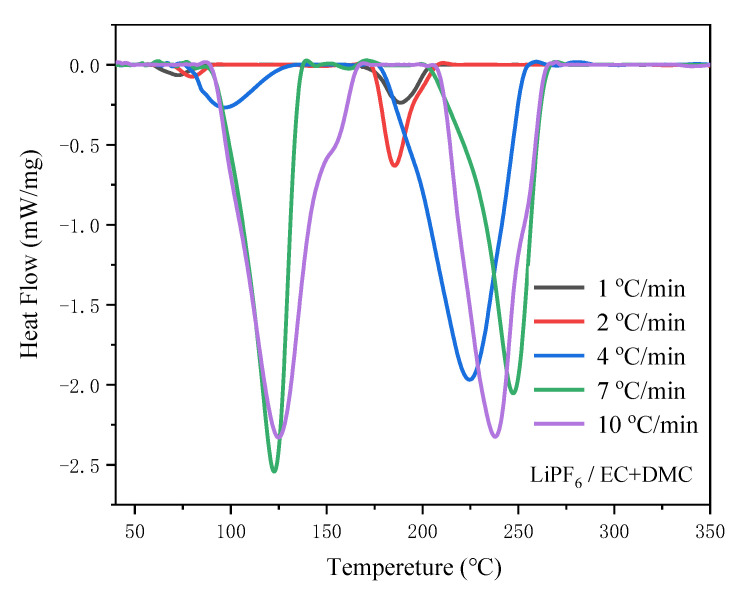
DSC curves of 1 M LiPF_6_/EC + DMC (2:3, *v*/*v*) at five different *β*.

**Figure 2:**
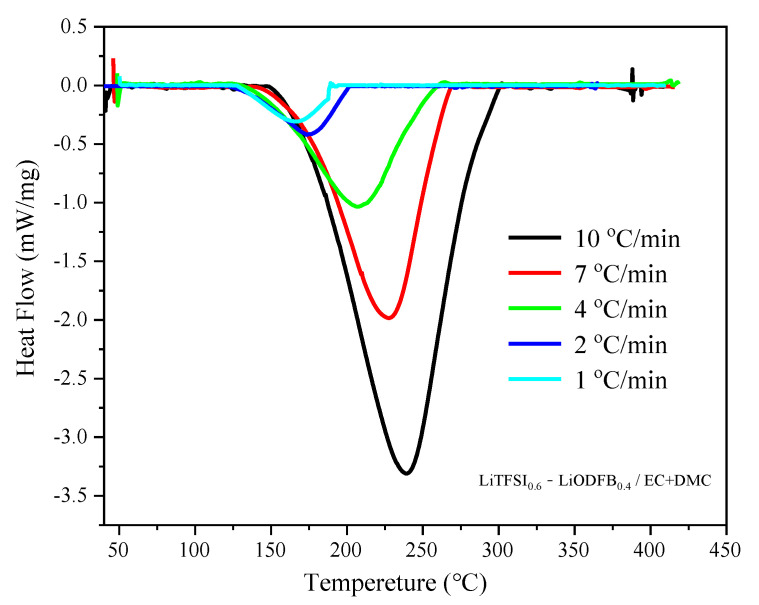
DSC curves of LiTFSI_0.6_-LiODFB_0.4_/EC + DMC (2:3, *v*/*v*) at five different *β*.

Figure 1 and Figure 2 respectively show the DSC curves of the 1 M LiPF_6_/EC + DMC (2:3, *v*/*v*) electrolyte and LiTFSI_0.6_-LiODFB_0.4_/EC + DMC (2:3, *v*/*v*) electrolyte at five different *β*. Table 2 summarizes the results of the *T*_o_, *T*_p_, and *T*_e_ decomposition temperatures. It can be seen that when *β* increased from 1 to 10 °C/min, the three decomposition temperatures in three endothermic curves of two electrolytes curves also rose. As the heating rate increased, the initial reaction temperature also increased, and the heat absorbed by the reaction was also enhanced. When *β* value was high, the system temperature rose rapidly over time, so a higher temperature was required to start the reaction. Nevertheless, once the reaction started, it was much faster than that at low heating rates, so *β* could greatly affect the thermal stability electrolyte parameters [40].

The LiPF_6_ carbonate electrolyte DSC curves included two endothermic peaks at *β* of 10 °C/min; the first one occurred from 89.3 to 167.3 °C and the second from 206.7 to 265.3 °C. The first peak began at 89.3 °C, corresponding to the decomposition of LiPF_6_, as shown in Equation (3) [41], and the moisture in the electrolyte accelerated the decomposition reaction.

(3) $${\mathrm{LiPF}}_{6}\left(s\right)⇌\mathrm{LiF}\left(s\right)+{\mathrm{PF}}_{5}\left(g\right)$$

The strong PF_5_ Lewis acid promoted the ring-opening polymerization reaction of low volatile solvents, and the low volatile compounds may be oligomers of the polyether carbonate in the thermal reaction (as shown in Equation (4)) [42,43]. The decomposition products of LiPF_6_ reacted with organic solvents, and solvents decomposed when the temperature exceeded 206.7 °C, resulting in the second endothermic peak [27].

(4) $$\mathrm{EC}\to {\left[{\left({\mathrm{CH}}_{2}{\mathrm{CH}}_{2}O\right)}_{n}\mathrm{COO}\right]}_{m}+{\mathrm{CO}}_{2}$$

LiTFSI-LiODFB dual-salt carbonate electrolyte was stable at low temperature. When the temperature exceeded 138.5 °C, the solvents began to decompose, and then the lithium salts decomposed successively [34]. The above analysis results show that LiTFSI-LiODFB dual-salt carbonate electrolyte has better thermal stability and a significantly greater thermal decomposition temperature than LiPF_6_ electrolyte.

#### 3.1.2. Thermal Decomposition Analysis by ARC

**Figure 3:**
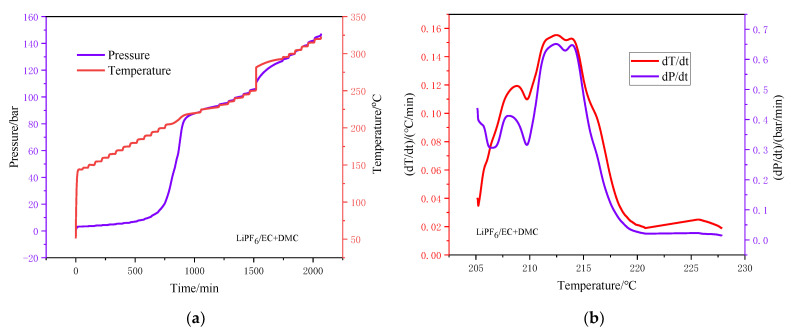
ARC curves of 1 M LiPF_6_/EC + DMC (2:3, *v*/*v*). (**a**) Pressure and temperature versus time curves; (**b**) Self-temperature rise rate and self-pressure rise rate versus temperature curves.

**Figure 4:**
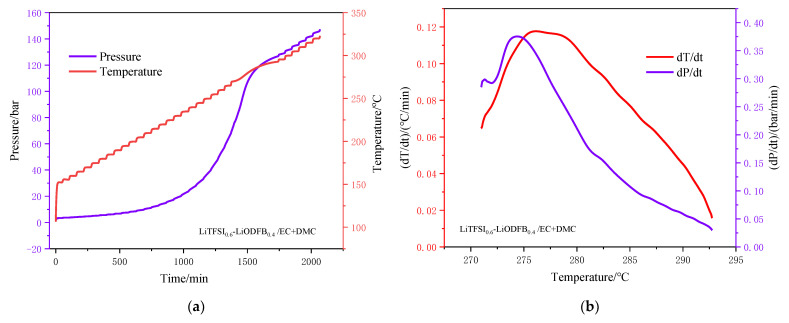
ARC curves of LiTFSI_0.6_-LiODFB_0.4_/EC + DMC (2:3, *v*/*v*). (**a**) Pressure and temperature versus time curves; (**b**)Self-temperature rise rate and self-pressure rise rate versus temperature curves.

Figure 3 and Figure 4 respectively show the thermal behavior of the two electrolytes in the ARC test, including the curves of temperature and pressure versus time as well as the self-temperature rise rate and self-pressure rise rate versus temperature. The characteristic parameters of the electrolyte ARC experiment are listed in Table 3, including the sample quality (ms), initial exothermic temperature (*T*_o,s_), end exothermic temperature (*T*_e,s_), maximum temperature rise rate (d*T*/d*t*)_max_, maximum pressure rise rate (d*P*/d*t*)_max_, and the temperature *T*_tm_, *T*_pm_ when the maximum temperature and pressure rise rate was obtained [35].

Figure 3 shows the pressure rise of the LiPF_6_-based electrolyte, which corresponds to two temperature ranges: 205.1–220.7 °C and 225.6–227.8 °C. The onset (205.1 °C) for an exothermic reaction was observed for the LiPF_6_ electrolyte. Figure 3b shows self-temperature rise rate with a maximum value of 0.155 °C/min and self-pressure rise rate with a maximum value of 0.65 bar/min. It is illustrated in Figure 1 that the exothermic reactions of LiPF_6_ started at 205.1 °C, which can be attributed not only to the release of PF_5_ from the PF_6_^−^(Equation (3)) but also the ring-opening polymerization reaction of EC and DMC (Equations (5) and (6)) [41,44]. The occurrence of this elimination explains the loss of condensed material during the reaction.

(5) $$R-O-\mathrm{CO}-O-R+F^{-}\to R-O-\mathrm{CO}-O^{-}+R-F$$

(6) $$R-O-\mathrm{CO}-O^{-}\to R-O^{-}+{\mathrm{CO}}_{2}$$

Figure 3a shows that the self-pressure rise rate reached the peak at 212.4 °C. However, the curve of self-temperature rise rate showed no peak before 212.5 °C. These results imply that most of the PF^−^ still existed and was stable at temperatures below 212.5 °C. From self-temperature rise rate and self-pressure rise rate curves of the two electrolytes, it can be found that the (d*T*/d*t*)_max_ and (d*P*/d*t*)_max_ of the LiTFSI-LiODFB electrolyte were lower than that of the LiPF_6_ electrolyte. As diagramed in Figure 4a, the electrolyte pressure began to rise before the exotherm. This explains why the endothermic heat of solvent decomposition LiTFSI-LiODFB dual-salt did not begin to decompose until 271.0 °C. The decomposition temperature of the LiTFSI-LiODFB dual-salt carbonate electrolyte range was 271.0–292.7 °C (seen from Figure 4b). Therefore, DSC results correlated well with the ARC, showing higher thermal stability of LiTFSI-LiODFB dual-salt carbonate electrolyte than LiPF_6_ electrolyte.

### 3.2. Thermal Kinetic Analysis

#### 3.2.1. Starink Method for Electrolyte *E*_a_ Calculation

**Figure 5:**
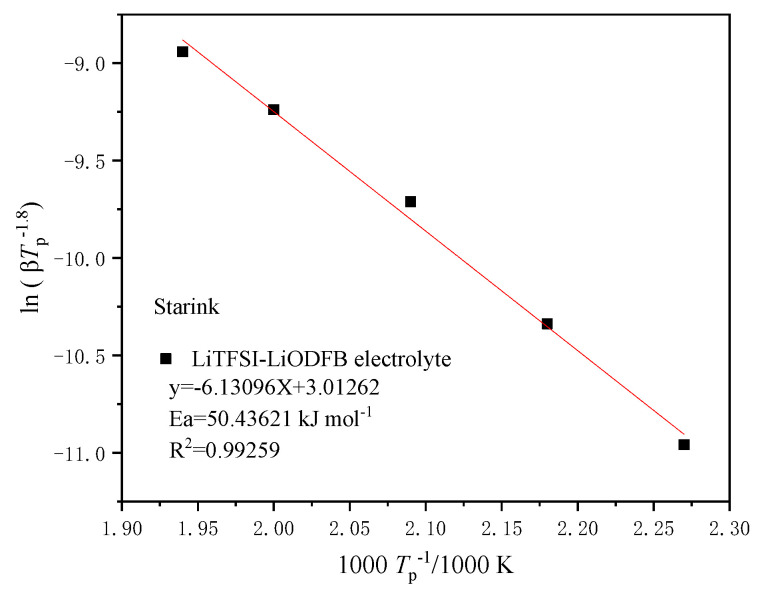
*E*_a_ plots of Starink model at different *β* in DSC experiments for the LiTFSI-LiODFB electrolyte.

Figure 5 illustrates the kinetic fitting curve of LiTFSI-LiODFB dual-salt carbonate electrolyte samplein the Starink method based on DSC experiments. It shows the lines obtained by fitting ln(1/*T*^1.8^) and 1000/*T* at different *β* (1, 2, 4, 7, and 10 °C/min). It can be calculated that dual-salt electrolyte *E*_a_ was 50.43 kJ/mol, and *R*^2^ was 0.9925.

#### 3.2.2. FWO Method for Electrolyte E_a_ Calculation

**Figure 6:**
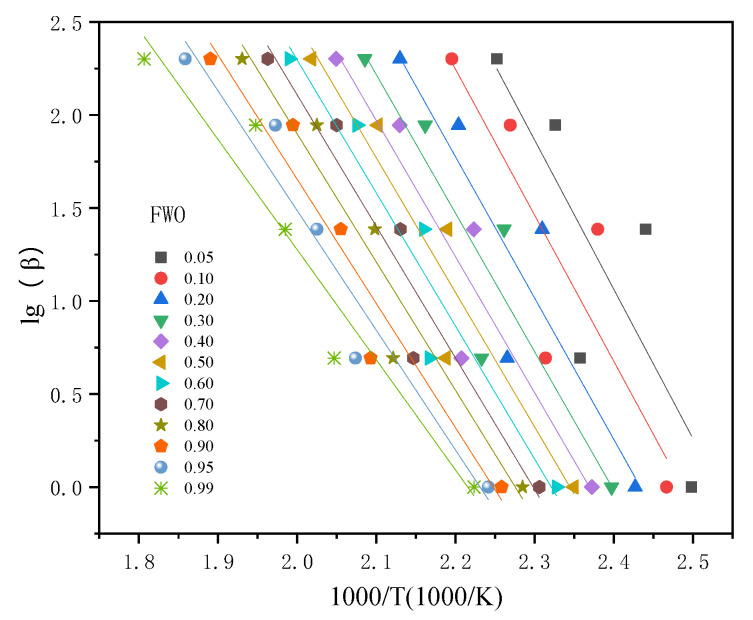
*E*_a_ fitting curves of the LiTFSI-LiODFB electrolyte by FWO method for conversion degree from 0.10 to 0.95.

The FWO model was adopted to further verify the *E*_a_. The fitting results of LiTFSI-LiODFB electrolyte in different conversion intervals (α, α= 0.05, 0.1, 0.2, 0.3, 0.4, 0.5, 0.6, 0.7, 0.8, 0.9, 0.95, and 0.99) are shown in Figure 6. The *E*_a_ for all the samples was calculated from the slope of the lines within the conversion range of 0.05–0.99. Among them, the fitting degree was lower, and the calculated *E*_a_ values were relatively higher than others in α  (0.05, 0.1, 0.2, 0.3, and 0.4), which could be attributed to the unstable premier reaction of pyrolysis. These fitted parallel straight-line plots indicated a slight change in the *E*_a_ values (46.8–63.4 kJ/mol) through the degradation processes. The average apparent activation energy ($E_{a}$) value was 56.39 kJ/mol, and *R*^2^ was 0.879, which is listed in Table 4.

#### 3.2.3. Thermokinetic Parameters Determined by Autocatalytic Model

According to the DSC curves of the LiTFSI-LiODFB electrolyte (Figure 2), the curves of the initial stage of the endothermic process did not overlap, and the entire spectrum was biased toward the high-temperature side. According to the empirical judgment method of the spectrum, it was preliminarily obtained that the endothermic process of the LiTFSI-LiODFB electrolyte was autocatalytic. The following reaction scheme was considered in Equations ((7)–(9)) [45]:

(7) A+nB⇌(n+1)B

(8) A⇌B

(9) B→C

This type of reaction generally accelerates as the reactant is consumed, and an autocatalytic substance is produced. The autocatalysis model is shown in Equation (10),

(10) $$\frac{d\alpha }{dt}=K_{0}e^{-E_{a}/RT}{\left(1-\alpha \right)}^{n1}\;\left(z+\alpha^{n2}\right)$$

where *n*1 and *n*2 respectively represent the first and second stages of the reaction, and *z* is the autocatalytic factor.

**Figure 7:**
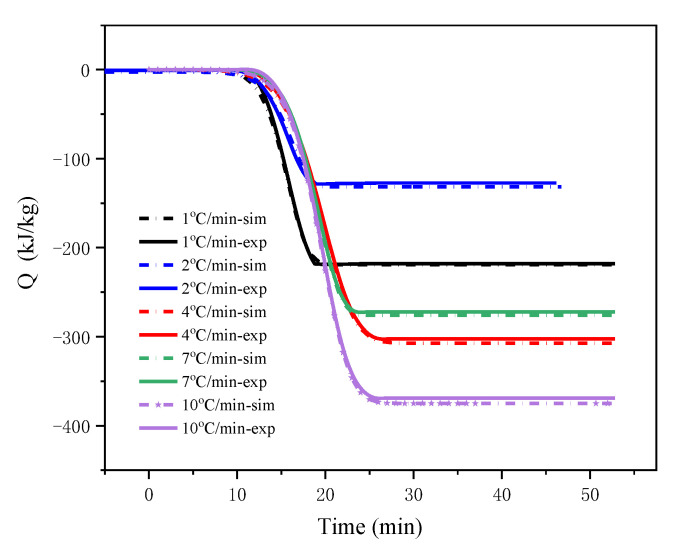
Evolution of heating production of LiTFSI-LiODFB electrolyte thermal decomposition reaction with time in experiment and simulation.

**Figure 8:**
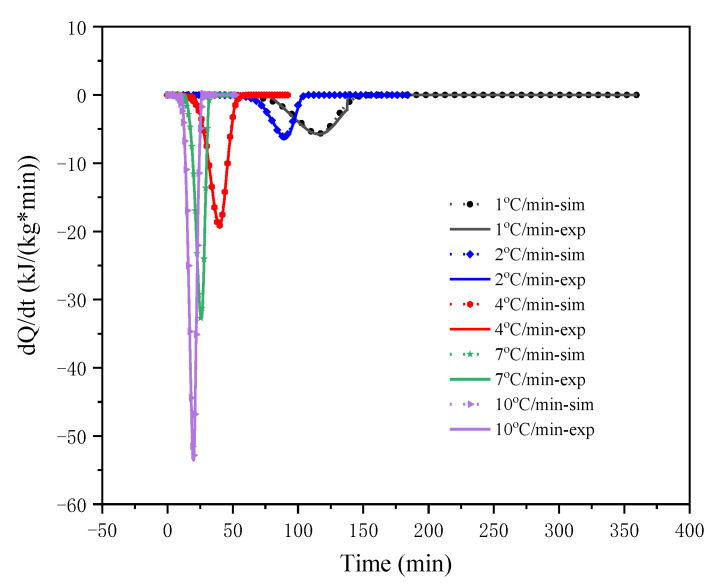
Evolution of heating production rate of LiTFSI-LiODFB electrolyte thermal decomposition reaction with time in experiment and simulation.

At different *β* (1, 2, 4, 7, and 10 °C/min), the relationship between heat release and time as well as the relationship between heat release rate and time are shown in Figure 7 and Figure 8, where *sim* and *exp* represent the simulation and the experimental data, respectively. It can be seen from the figure that the fitting results of the autocatalytic model and the DSC experimental data were mostly completely scattered on the same line, and the simulation results had ideal consistency. The calculation results of the dynamic parameters are listed in Table 4. The *E*_a_ obtained by the autocatalysis model fitting was 52.93 kJ/mol. The comparison shows that the kinetic parameters simulated by the autocatalysis model were roughly the same as those calculated by the isoconversional method.

## 4. Conclusions

1. The thermal behavior tests of the LiPF_6_ and LiTFSI-LiODFB dual-salt carbonate electrolyte by ARC and DSC indicated that the latter had better thermal stability. At 89.3 °C, the LiPF_6_ carbonate electrolyte conductive salt and moisture undergo an endothermic decomposition reaction to generate strong Lewis acids PF_5_, LiF, and trace moisture, accelerating the decomposition reaction. When the temperature exceeded 206.7 °C, the strong PF_5_ Lewis acid promoted the ring-opening polymerization reaction of low-volatility solvents, and the decomposition products of LiPF_6_ reacted with organic solvents. While LiTFSI-LiODFB dual-salt carbonate electrolyte was stable below 138.5 °C, the solvents began to decompose. The lithium salts decomposed successively when the temperature exceeded 271.0 °C.
2. Starink, FWO kinetic models, and autocatalytic methods were used to calculate the *E*_a_ values of LiTFSI-LiODFB dual-salt electrolyte. The results showed that the values determined by the three methods were similar, with an average value of 53.25 kJ/mol. According to the simulation results, the mixed salt is considered to follow the autocatalytic model. The findings can provide a reference for the future application of dual-salt in different types of new lithium-ion batteries.

## Figures and Tables

**Table 1:** Physico-chemical properties of electrolyte lithium salts and solvents.

Compounds	Chemical Formula	Boiling Point (°C)	Melting Point (°C)
EC	C_3_H_4_O_3_	248	38
DMC	C_3_H_6_O_3_	91	0.5
LiTFSI	C_2_F_6_LiNO_4_S_2_	190.5	234–238
LiODFB	C_2_BF_2_LiO_4_	–	–
LiPF_6_	LiPF_6_	–	200

**Table 2:** Characteristic temperature of electrolytes in the DSC experiment at different *β*.

Heating Rate (°C/min)	LiPF_6_/EC + DMC						LiTFSI_0.6_-LiODFB_0.4_/EC + DMC		
	Stage I (°C)			Stage II (°C)					
	*T*_0_	*T*_p_	*T*_e_	*T*_0_	*T*_p_	*T*_e_	*T*_0_	*T*_p_	*T*_e_
1	60.9	72.3	88.1	167.6	188.5	209.4	113.7	167.2	185.5
2	67.7	81.33	92.0	173.3	205.3	208.0	108.0	185.6	223.6
4	76.0	99.7	137.0	176.7	224.6	255.3	110.0	204.8	242.4
7	79.5	123.9	137.9	201.3	247.5	266.7	129.6	226.4	254.5
10	89.3	125.0	167.3	206.7	253.3	265.3	138.5	243.5	294.0

**Table 3:** Characteristic parameters of electrolyte samples in the ARC experiment.

Sample	m_s_ (g)	*T*_o,s_ (°C)	*T*_e,s_ (°C)	(d*T*/d*t*)_max_ (°C/min)	*T*_tem_ (°C)	(d*P*/d*t*)_max_ (bar/min)	*T*_pre_ (°C)
LiPF_6_/EC +DMC	2.311	205.1 ^1^, 225.6 ^2^	220.7 ^1^, 227.84 ^2^	0.15526	212.5	0.6510	212.4
LiTFSI_0.6_-LiODFB_0.4_/EC+ DMC	2.55	271.0	292.7	0.117	276.1	0.3754	274.3

^1^ Represents the exothermic phenomenon detected by the ARC experiment for the first time, while ^2^ represents the second time.

**Table 4:** Thermokinetic parameters of LiTFSI-LiODFB dual-salt electrolyte calculated by different kinetic methods and simulation.

	Methods	Starink	FWO	Autocatalytic Model
Parameter				
*E*_a_ (kJ/mol)		50.43	56.39	52.93
*R* ^2^		0.992	0.879	‒
lnA		‒	‒	7.8

## Data Availability

The data presented in this study are available on request from the corresponding author.
